# The strategic use of embolization in treating infantile fibrosarcoma-related heart failure: a case report

**DOI:** 10.3389/fsurg.2025.1638718

**Published:** 2025-10-30

**Authors:** M. E. Bartoli, G. Cassanelli, G. L. Natali

**Affiliations:** 1 Pediatric Surgery Academy, Tor Vergata University of Rome, Rome, Lazio, Italy; 2 Interventional Radiology Unit, Bambino Gesù Children's Hospital- IRCCS, Rome, Lazio, Italy

**Keywords:** infantile fibrosarcoma (IFS), high-flow heart failure (HFHF), arteriography, embolization, case report

## Abstract

**Introduction:**

Infantile fibrosarcoma (IFS) represents the most common non-rhabdomyosarcoma soft tissue tumor, with 80% of diagnoses under the first year of life. In contrast with adult fibrosarcoma, IFS has lower risks of metastasis, better long-term survival rate, and higher chemosensitivity. Conservative surgery, in association with chemoradiotherapy in case of metastasis or recurrence, usually represents the gold standard treatment.

**Case:**

We examined the case of a 2-month-old female patient affected by retroperitoneal congenital fibrosarcoma, which had caused high-flow heart failure (HFHF) due to its hypervascularization and multiple arteriovenous fistulas. Given the complexity of the case and its atypical vascularization, after multidisciplinary discussion, we decided to perform an endovascular approach rather than a surgical one, aiming to interrupt pathological flow to this abdominal mass. The procedure was well tolerated with fast improvement in both clinical and ultrasound markers of heart failure.

**Conclusion:**

This is the first instance of arteriographic application for the management of HFHF caused by hypervascularized retroperitoneal IFS that we are aware of. In conclusion, we advise using this approach because of its safety and effectiveness, even though it necessitates a high level of experience.

## Introduction

Infantile fibrosarcoma (IFS), a low-grade nonrhabdomyosarcoma soft tissue sarcoma (NRSTS), is the most prevalent soft tissue sarcoma in children under one year of age (1).

It can appear during the first five years of life or be present from birth, particularly in children younger than two (2).

This tumor is typically axial (20%) or limb-related (71%). Other localizations, such as the tongue and oral cavity, ovary, retroperitoneum, chest wall, heart, and bowel, are rarely reported (3).

IFS is distinguished from adult fibrosarcoma by having a higher long-term survival rate (90% at 5 years), a lower incidence of metastasis (<10%), and greater chemosensitivity (2, 4).

The clinical presentation is usually that of a bulging, rapidly growing mass on the extremities or trunk (5). This tumor tends to be locally invasive and metastasizes infrequently (6).

According to the literature, a spontaneous regression of IFS of the left forearm had been described (7).

Current treatment for IFS includes initial biopsy and chemotherapy, followed by conservative resection when the tumor shrinks (8, 9).

A multidisciplinary approach is necessary for approximately 48%–62% of primary tumors that cannot be removed, including local radiotherapy in certain circumstances and preoperative cytoreductive treatment (10).

Conservative resection with negative surgical margins is the key to avoid recurrence (6), although it can lead to significant long-term sequelae due to radical or mutilating surgery (11).

Neoadjuvant chemotherapy with alkylating agents such as vincristine, dactinomycin, and cyclophosphamide (VAC) is used to reduce the volume of the tumor before surgical resection.

It is advised that patients with macroscopic residual disease receive postoperative chemotherapy as their first line of treatment in order to reduce local recurrence (10).

To avoid their cytotoxic effects, new targeted therapies have been proposed considering the molecular biology of the tumor: in particular, the use of tropomyosin-related kinase inhibitors has been described as therapeutic options in neoadjuvant, adjuvant, or metastatic settings (12).

Here, we presented a fascinating case of a 2-month-old patient with congenital fibrosarcoma who came to our attention with cyanosis and respiratory distress as a result of an intralesional venous-arteriosus shunt that was hyperinflowing to the inferior vena cava (IVC).

## Case

A 20-day-old female who was born at 38 weeks gestational age was diagnosed with an abdominal mass during pregnancy and brought to our institution.

Clinically, there was no sloping edema or respiratory distress; blood pressure and urine output were both normal. No congenital heart-related conditions were found on the echocardiogram.

Following a multidisciplinary consultation, she underwent thoraco-abdominal CT scan, abdominal ultrasound, and was tested for tumoral markers (Alpha-fetoprotein (AFP), Clinostatic Renin, Human Chorionic Gonadotropin (hCG), Chromogranin, Carcinoembryonic Antigen (CEA), Urinary Vanillylmandelic Acid (VMA) and Homovanillic Acid (HVA), Ferritin and Transferrin).

Abdominal ultrasound revealed a retroperitoneal expansive mass, encasing iliac, renal, and abdominal aortic vessels, with hypervascularization at Doppler-US.

A CT scan confirmed the presence of this bulky mass, measured in 50 × 43 × 27 mm (a total volume of approximately 30.382 mm^3^), which caused displacement of the right kidney and compression of the inferior vena cava, consequently severely dilated.

A month later, an MRI of the abdomen revealed an enlarged mass (70 × 45 × 30 mm).

Since there were no neurological symptoms like sensory or motor deficits and there were no signs of spinal canal invasion at the CT scan, we did not perform an MRI of the cerebrospinal canal.

It is important to underline that CT scan was carried out before MRI because, using specific contrast agents, it would have provided a faster evaluation of the mass size, shape, and relationship with blood vessels; furthermore, it would have better identified calcifications or necrotic areas within the tumor, which are a common finding in IFS.

With the exception of an increase in TSH and normal FT3 and FT4 values that did not appear to be connected to the lesion, all tumoral markers were negative.

Given the close proximity to great abdominal vessels, a percutaneous bioptical approach was not feasible, necessitating a surgical biopsy of the lesion.

A congenital fibrosarcoma with PRKAR1B::BRAF fusion was found by histological analysis: PRKAR1B encodes a regular subunit of the cyclin AMP-dependent protein kinase A complex, implicated in neurodegenerative dementia but not well associated with cancer, while B-RAF is a well-known proto-oncogene (13).

This implied that Vemurafenib, a B-RAF inhibitor, might be required for targeted treatment.

**Figure 1:**
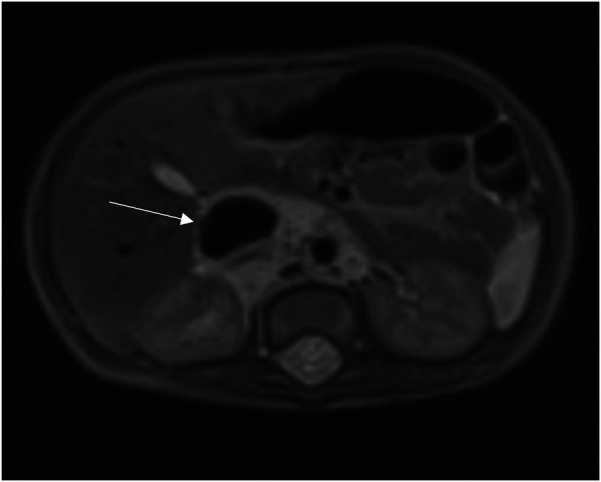
Dilated Inferior Vena Cava (IVC) on axial section at MRI.

**Figure 2:**
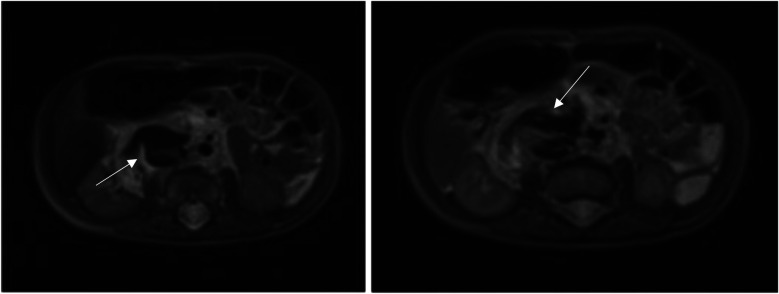
Intralesional arteriovenous shunt on axial section at MRI.

In order to better evaluate the vascularity and vessel proximity of the lesion, our oncologists suggested an abdominal MRI. An intralesional arteriovenous shunt was found in conjunction with a significant IVC dilatation brought on by hyperinflow (Figures 1, 2).

After a month, the patient was readmitted with cyanosis and severe respiratory distress.

Anticoagulant therapy was started after atrio-caval thrombosis diagnosed by Chest-CT.

When our cardiologist examined the patient, he discovered signs of systemic overflow-induced cardiomegaly and high-flow heart failure (Right Ventricle Pressure, RVP = 60 mmHg, 2/3 of Systemic Pressure, SP).

Diuretic and vasodilator therapy with furosemide and milrinone was started in consideration of clinical signs of heart failure and elevated NT-pro-BNP levels (up to 8.385 pg/ml).

Indicators of pulmonary hypertension were monitored.

Given the high risk of heart failure persistence, we chose to use digital-subtracted angiography (DSA) to embolize vascular afferences to the lesion: via right femoral access, we performed an arteriography that demonstrated many arteriovenous fistulas inside the lesion with many lumbar and iliac vessels draining into IVC.

**Figure 3:**
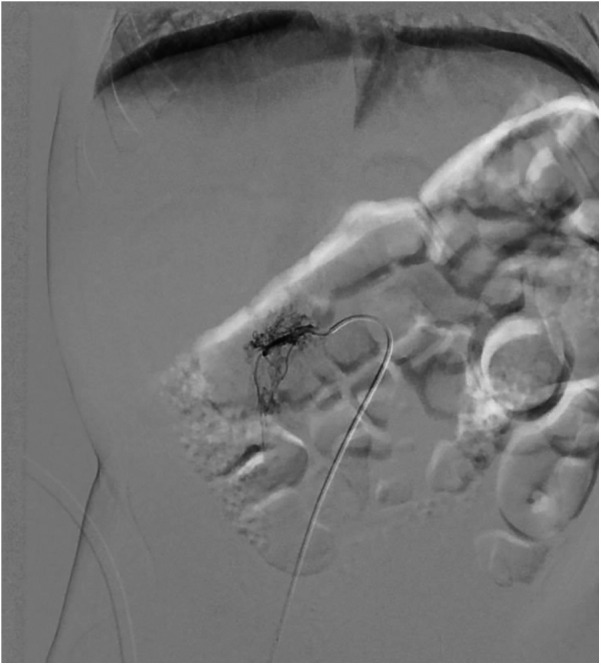
Coil embolization of hypertrophic lumbar vessel afferent to the lesion.

A single hypertrophic lumbar vessel was successfully selectively catheterized and embolized using 2 Concerto coils (2 mm × 4 cm and 2 mm × 6 cm, Figure 3).

Three days after the procedure, which was well tolerated, indirect signs of heart failure decreased (NT-pro-BNP dropped from 8.385 pg/ml to 2.068 pg/ml).

US and clinical settings improved: in particular, six days after the procedure, RVP was approximately 50 mmHg (down from 60 mmHg).

Additionally, about two weeks after embolization, she began off-label targeted immunotherapy with oral Vemurafenib at a dose of 10 mg/kg twice a day.

An echocardiogram performed about a month after embolization revealed good heart function and cardiocirculatory compensation (Ejection function of left ventricle 65%), with indirect signs of normal pressure gradient in the pulmonary valve (≤25 mmHg), which was secondary to the prior high right heart preload.

**Figure 4:**
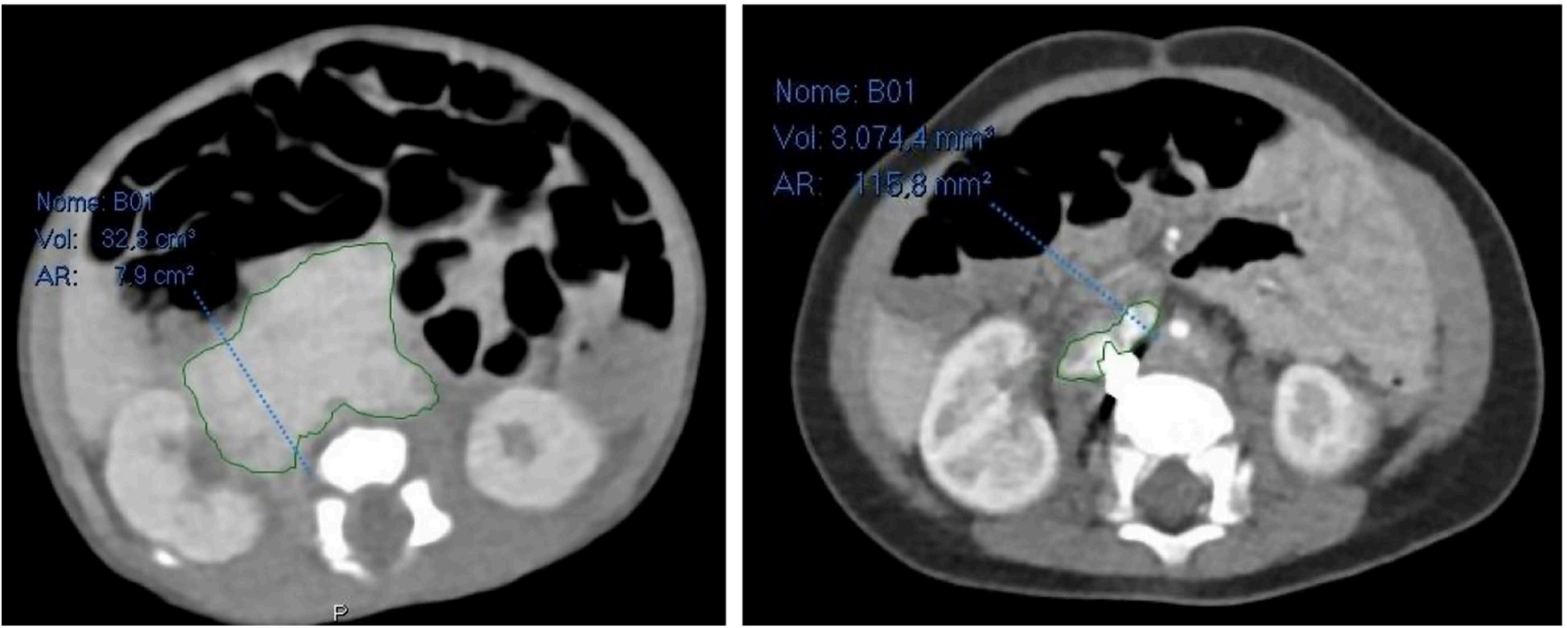
On the left abdominal CT scan at admission, on the right abdominal CT scan 6 months after embolization.

Furthermore, 6 months after embolization, a CT scan revealed that the retroperitoneal mass had significantly shrunk in comparison to previous radiological exams: it measured 30.2 × 14 × 36 mm (a total volume of approximately 7.965 mm^3^, about 26,2% of the original mass), and it was difficult to determine whether it was still surrounding retroperitoneal vessels (Figure 4).

The oncological follow-up is still ongoing.

## Discussion

We presented the case of a rare localization of congenital fibrosarcoma in the retroperitoneum.

This anatomical specific localization represents an overall challenge, both clinical and surgical, because chemotherapeutic drugs have difficulty reaching it and it could not be surgically approached due to its close proximity to the great abdominal vessels.

The EpSSG recommends conservative tumor resection for localized disease and vincristine-actinomycin (VA) chemotherapy as the first-line option for patients with unresectable disease (14).

Neoadjuvant chemotherapy has been recommended in some cases in order to minimize the need for mutilating resections (15, 16).

Recent years have seen the development of novel targeted therapies, particularly tropomyosin receptor kinase (TRK) inhibitors like Larotrectinib, which have been successfully used to stop or prevent tumor growth thanks to their ability to block the tyrosine kinase domain of TRK protein that is constitutively activated in IFS and other sarcomas with overall response rates above 90% (17–19).

70% of cases carry the ETV6-NTRK3 gene fusion as an oncogenic driver (20, 21).

Patients with TRK fusion sarcomas might proceed to surgery after treatment with this medication, thereby avoiding demolitive surgery in case of insufficient response to chemotherapy or in case of metastatic disease (14, 17).

However, the use of this medication was not feasible in this patient due to a PRKAR1B::BRAF fusion.

This kind of mutation is uncommon in IFS but had been described by Charo et al. in 2018 in a pregnant 32-year-old woman affected by a mid-jejunal gastrointestinal stromal tumor (GIST), which was resected, but, considering its teratogenic potential, a therapy with a B-RAF inhibitor like Imatinib or Vemurafenib was not recommended (13).

In this instance, however, given the patient's age, our oncologists determined there were no contraindications to using Vemurafenib off-label. Its safety and efficacy should be assessed during her oncological follow-up in the upcoming months.

The additional issues in this case were represented by the hypervascularization of this abdominal mass and, more importantly, by the arteriovenous fistulas between the arteries feeding the lesion and the IVC with subsequent right heart failure due to overflow.

IFS is usually fed by arteries of irregular caliber in a disordered branching pattern; the venous phase shows several tortuous and slightly enlarged veins, while the capillary phase is characterized by a dense but inhomogeneous tumor blush (22).

In order to treat this condition, in accordance with neonatal surgeons and oncologists, we decided to perform an abdominal arteriography in order to embolize the pathological hypertrophic vessels inside the fibrosarcoma, blocking its blood supply and causing its shrinkage before eventual surgery.

To the best of our knowledge, this is the first description of embolization of a retroperitoneal fibrosarcoma in an infant: in 2021 Filho et al. described a successful transarterial chemoembolization (TACE) in a 71-year-old patient with an inoperable retroperitoneal soft tissue sarcoma (23).

Significant tumor necrosis and symptomatic relief were the outcomes of this intervention, underscoring the potential use of interventional radiology as a bridging or palliative treatment in situations where surgery is not practical.

Embolization offers several advantages: firstly, it targets the tumor precisely, reducing damage to surrounding structures. Secondly, by cutting off the tumor's blood supply, debulking of the tumor are expected, making surgery easier.

Even if the endovascular approach is a relatively safe technique, it presents some limitations: it may not always be feasible and may require great technical expertise, so it should be managed by dedicated pediatric interventional radiologists.

## Conclusion

Tumoral embolization is a promising approach for congenital fibrosarcoma; however, more research and cooperation between a multidisciplinary team of surgeons, interventional radiologists, and oncologists are required to determine its feasibility and medium- and long-term efficacy in pediatric age.

## Data Availability

The raw data supporting the conclusions of this article will be made available by the authors, without undue reservation.
